# The effects of involving a nurse practitioner in primary care for adult patients with urinary incontinence: *The PromoCon study (Promoting Continence)*

**DOI:** 10.1186/1472-6963-8-84

**Published:** 2008-04-15

**Authors:** Pytha Albers-Heitner, Bary Berghmans, Manuela Joore, Toine Lagro-Janssen, Johan Severens, Fred Nieman, Ron Winkens

**Affiliations:** 1 Integrated Care Unit, University Hospital Maastricht, Maastricht, The Netherlands; 2 Pelvic care Center Maastricht (PcCM), University Hospital Maastricht, Maastricht, The Netherlands; 3 Department of Clinical Epidemiology and Medical Technology Assessment, University Hospital Maastricht, Maastricht, The Netherlands; 4 Women's Studies Medicine, Department of General Practice UMC, St. Radboud University Hospital Nijmegen, Nijmegen, The Netherlands; 5 Department of Health Organisation, Policy, and Economics, Maastricht University, Maastricht, The Netherlands; 6 Department of General Practice, Maastricht University, Maastricht, The Netherlands

## Abstract

**Background:**

Urinary incontinence affects approximately 5% (800.000) of the Dutch population. Guidelines recommend pelvic floor muscle/bladder training for most patients. Unfortunately, general practitioners use this training only incidentally, but prescribe incontinence pads. Over 50% of patients get such pads, costing €160 million each year. Due to ageing of the population a further increase of expenses is expected. Several national reports recommend to involve nurse specialists to support general practitioners and improve patient care. The main objective of our study is to investigate the effectiveness and cost-effectiveness of involving nurse specialists in primary care for urinary incontinence. This paper describes the study protocol.

**Methods/Design:**

In a pragmatic prospective multi centre two-armed randomized controlled trial in the Netherlands the availability and involvement for the general practitioners of a nurse specialist will be compared with usual care. All consecutive patients consulting their general practitioner within 1 year for urinary incontinence and patients already diagnosed with urinary incontinence are eligible. Included patients will be followed for 12 months.

Primary outcome is severity of urinary incontinence (measured with the International Consultation on Incontinence Questionnaire Short Form (ICIQ-UI SF)). Based on ICIQ-UI SF outcome data the number of patients needed to include is 350. For the economic evaluation quality of life and costs will be measured alongside the clinical trial. For the longer term extrapolation of the economic evaluation a Markov modelling approach will be used.

**Discussion/Conclusion:**

This is, to our knowledge, the first trial on care for patients with urinary incontinence in primary care that includes a full economic evaluation and cost-effectiveness modelling exercise from the societal perspective. If this intervention proves to be effective and cost-effective, implementation of this intervention is considered and anticipated.

**Trial registration:**

Current Controlled Trials ISRCTN62722772

## Background

### Problem definition

#### The disease

Approximately 5% (800.000 people) of the Dutch population suffer from urinary incontinence (UI) [1]. This concerns predominantly women, and prevalence increases with age [2]. Among patients aged over 75, the prevalence rises up to 30%. UI is infamous for its impact on general well being and social activities [3,4]. If not treated, UI is a chronic, not self-limiting disorder with a strong tendency to worsen over time. It is one of the most important reasons for institutionalisation of elderly people in nursing homes.

#### The health care problem

In the Netherlands, annually €160 million is spent on incontinence pads [1]. Considering the ageing of the population a further increase in these expenditures is expected [5-7]. Guidelines indicate that for most patients with UI pelvic floor muscle and/or bladder training is the best non-invasive treatment to solve the problem, rather than just compensate the urine loss [2,5,6,8,9]. To date, despite guidelines, training is used only incidentally by general practitioners (GPs), probably because it is too time consuming [5,6,8,10-12]. Most GPs choose an easier, but non-curative and ultimately far more expensive, alternative: prescribing incontinence pads. More than 50% of patients get incontinence pads, especially the elderly [1,12].

#### Usual care

For the vast majority of health problems in the Dutch population, the GP is the initial person to contact and is also the gatekeeper for specialist care. This also applies for UI. Most patients (± 96%) with UI are kept within primary care [9,13]. Only few patients (total < 5%) are referred to a specialist (in most cases a urologist or gynaecologist) or a physical therapist [5,13]. National guidelines on UI are available for primary care [9,14]. These guidelines are followed only incidentally, irrespective of the type of guideline or clinical problem involved [10-12,15]. A major reason why the guidelines are not used, may be because consultations take more time when following guidelines [5,16].

#### Motivation and relevance for the chosen intervention

In recent years, there is a growing emphasis in the Netherlands to involve nurse specialists in general practice with positive findings for chronic disorders [5,6,17,18]. Nurse specialists have specific expertise, and are capable of spending more time on motivating patients. Incorporating nurse specialists may offer a solution to the inadequate care for UI with inappropriate prescribing of incontinence pads. After special training, and using their specific expertise on UI, they are well equipped to support the GP after the initial problem definition by the GP. Their acceptance by patients and GPs, feasibility and usefulness in management, and effectiveness in treating UI have been reported [8,19-23]. However, to our knowledge, studies with a full economic analysis and cost-effectiveness modelling exercise from the societal perspective of involving nurse specialists for UI are not available.

### Objective

The main objective is to study whether the availability and involvement of a nurse specialist in a new role as a substitute for the GP in the management of UI in general practice leads to more efficient care for adult UI patients. We envision that the effects of the intervention will be such that care will improve, hence more patients can be treated effectively and therefore will use less incontinence pads, which leads to a reduction in costs.

The following research questions were formulated:

1. Does the availability and the involvement of a nurse specialist as a substitute for the GP for adult persons with UI lead to a reduction in the severity of UI compared to care as usual by the GP?

2. Does the involvement of such a nurse specialist lead to a better quality of life of the patients?

3. Is the involvement of such a nurse specialist cost-effective compared to care as usual by the GP?

4: Does the involvement of a nurse specialist on UI lead to a better satisfaction of patients, GPs and other health care providers?

5. To what extent do GPs use the nurse specialist?

6. What are reasons for the GPs for (not) using the nurse specialist? Does the availability of the nurse specialist change the perception of GPs about the treatment via the nurse specialist?

## Methods/Design

**Figure 1:**
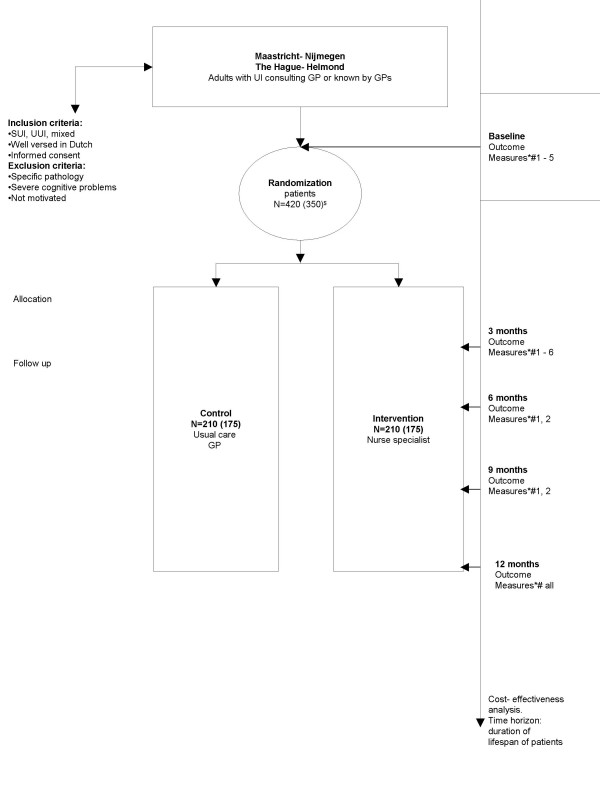
**Study design**. * Primary outcome measure: • severity urinary incontinence (ICIQ-UI SF) **# Secundary outcome measures: **1. health care and productivity, time, travel costs. 2. generic QOL: EuroQol. 3. incontinence specific QOL: IIQ-7. 4. quantification of symptoms: bladder diary. 5. satisfaction of patients. 6. perceptions GPs and nurse specialists. $ needed number with and without drop-out/loss-to-follow up

This pragmatic prospective multi-centre two-armed randomized controlled trial is now conducted among patients with UI in general practices in four regions in the Netherlands. Initially, the trial was planned to be conducted in at least two regions (Maastricht and Nijmegen). During the preparation of the trial expert key persons on UI in the regions of Helmond and The Hague expressed their willingness to participate as well (see Figure 1). The study protocol is approved by the Medical Ethical Committees of all the involved medical centres and hospitals. Patients will be randomized into an experimental group with availability of care by the nurse specialist or a control group receiving the usual care from their GP. Included patients will be followed for 12 months.

The randomization process is concealed from those responsible for recruiting patients using central telephone randomization. Because only a small number of patients is to be expected per GP, patients are randomized using a separate randomization list for each participating region.

Blinding patients and health care providers is not possible. Personnel collecting outcome data are blinded to the patients treatment allocation. A patient in the control group can not be referred by the GP to use the route via the nurse specialist, to avoid contamination.

### Study population

#### Inclusion criteria

All consecutive patients consulting their GP for symptoms and signs of stress, urgency and mixed UI (according to the guidelines of the Dutch College of General Practitioners on UI [9]) for a period of one year and UI patients diagnosed as such by the GPs in the past are eligible for the study [9,24,25]. Patients who consult their GP for UI are actively recruited by the GP to participate in the study. Known UI patients are selected by the GP on eligibility and will be invited by the GP to participate in the study. In case of doubt by the GP on eligibility the patient will be invited for a consultation on UI by the GP. Regularly and repeatedly, GPs will be stimulated to include patients for the study. Information, reminders and newsletters are part of the strategy to enhance the GPs awareness of the availability of the nurse specialist and its potential benefits for this specific health care.

#### Exclusion criteria

Excluded are patients below 18, women with prolapse degree III or more, patients with signs of reflex- or overflow incontinence, tumours in the abdomen, severe neurological diseases associated with incontinence (multiple sclerosis, CVA, diabetes, cauda equina syndrome), actual urinary tract infection, hematuria without urinary tract infection, men below 65 with unexplained incontinence, patients with failure after operation or failure of conservative therapy during the past half year (or longer, provided there is no relapse which causes dissatisfaction with the present situation), severe cognitive problems, not well versed in Dutch, refusing to participate/cooperate, patients for whom the GP considers the management via the nurse specialist as impossible/undesired and UI patients in care and nursing homes.

#### Informed consent

After written informed consent all patients included have baseline data collection, urologic, obstetric and gynaecologic history in women and urologic history in men. All measurements in this study are performed by postal questionnaires managed by an independent research-assistant.

### Intervention(s)

**Figure 2:**
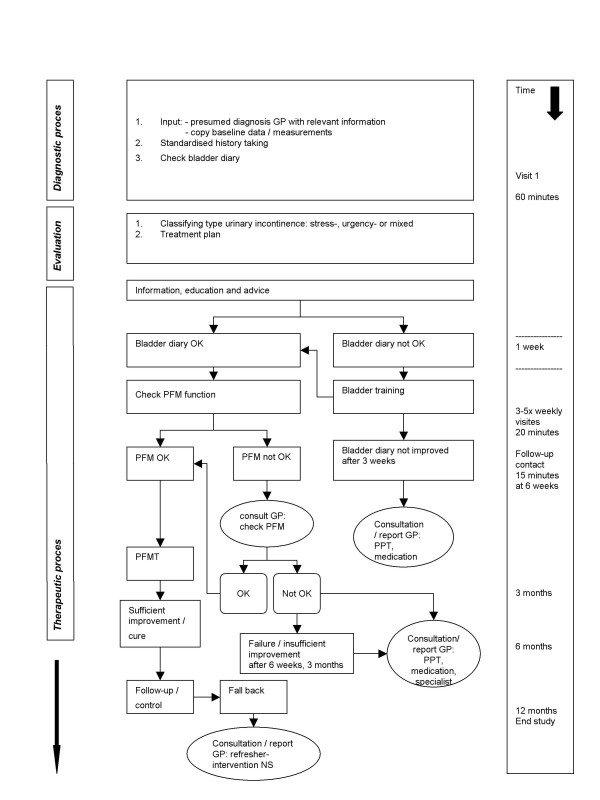
**Flow chart intervention nurse specialist for patients with UI**. PFM (T) = Pelvic Floor Muscle (Training); GP = general practitioner; PPT = pelvic physiotherapist; NS = nurse specialist. The used terminology is according to the definitions as recommended by the International Continence Society.

The intervention is designed as close as possible to treatment options in clinical practice, including 'cascades' of patient management choices (Figure 2). When the patient is allocated to the intervention group the GP refers the patient to the nurse specialist according to a care protocol. All participating GPs are personally informed about the demarcation and definition of job responsibilities and competence profile of the nurse specialist towards GPs and other health care professionals involved [26,27]. This information is also available to the GP as written information.

### Training and competences of the nurse specialist [see Additional file 1]

The training program for the nurse specialists how to support the GP, by taking care of the diagnostic and therapeutic management of patients with the most prevalent types of UI, is gradually developed during the preparation period of the study. All nurse specialists will have to prove their competences in an individual assessment.

### Intervention nurse specialist [see Additional files 2 and 3]

The main goal of the intervention of the nurse specialist is to provide a tailored, patient specific diagnostic and treatment plan, thereby preventing or reducing the use of incontinence pads. Based on guidelines and protocols the nurse specialist takes over tasks from the GP related to diagnostics, intervention and monitoring of patients with the most prevalent types of UI (stress, urgency and mixed UI) [28-31]. The GP keeps final responsibility. Furthermore, the nurse specialist supports patient motivation, compliance and adherence both on the short and the long term by monitoring patients over time in a systematic way. This ensures that patients will accept, understand, are willing and able to adhere to advices on lifestyle, bladder- and pelvic floor muscle training according to a health education model [22]. Another task of the nurse specialist is to give adequate information and advice about (when still necessary) the choice and the use of non-curative means like incontinence pads. She/he will always report to the GP and acts as the contact person between the other healthcare providers. In case of unclear pathology, a complex health problem or failure of treatment the nurse specialist can advice a referral to a specialist or specialised physical therapist. In all cases, the decision for referral is to be made by the GP. Altogether this means that the nurse specialist will report to the GP on each patient after first problem assessment and at the end of the intervention period. A regular meeting between nurse specialist and GP to discuss patients will be organised when needed.

### Outcome measurements and data collection

The primary outcome measure is the severity of involuntary loss of urine. This is measured by the self-completed condition specific International Consultation on Incontinence Questionnaire Short Form (ICIQ-UI SF) which measures frequency, volume and impact on daily life of involuntary urine loss [32,33]. The outcome is a sum score of the first two weighted items and the Visual Analogue Scale (VAS) score of impact on daily life. The questionnaire underwent extensive psychometric testing and is rated by the International Consultation on Incontinence (ICI) as Grade A, meaning highly recommendable [3].

Secondary outcome measures:

1. Generic quality of life is measured with the self-completed standardised EuroQol quality of life questionnaire (EQ-5D) [34]. It provides a simple descriptive profile, a single index value for health status and a VAS scale (0–100). Health states defined by the 5-dimensional descriptive system can be converted into a weighted health state index by applying scores from EQ-5D "value sets" elicited from general population samples.

2. Health care costs (the use of diagnostics, treatment and incontinence pads) and non-medical costs (productivity costs, time costs and travel costs) are collected using both registration systems and retrospective cost questionnaires. Because of recent literature and own experiences in similar trials the use of these questionnaires was favoured above cost diaries [35-37].

3. Condition-specific quality of life is measured with the self-completed International Incontinence Questionnaire (IIQ). For practical reasons we used the short form of this validated (7 items) questionnaire that measures impact of urinary loss on five domains: 'mobility', 'physical functioning', 'social functioning', 'emotional health' and 'embarrassment' [38,39].

4. Symptoms relevant for UI (the degree of pad usage, times of micturition, incontinence episodes, the degree of urgency, complications, complaints) is measured with a self-completed bladder diary during 3 consecutive days [40-43]. Considering the restricted diagnostic value of lower urinary tract symptoms (LUTS) for a final diagnosis (grade D) and to minimize complexity and to maximize compliance (Gordon 2001) [3] to registration of the bladder diary this registration will be restricted to a micturition time chart with the most relevant LUTS which give an impression of type, severity and impact of UI [3].

5. Patients' satisfaction with provided care for UI by the GP and/or the nurse specialist was originally planned to be measured with the for UI adjusted QUOTE self-completed questionnaire. This showed to be not suitable to answer our research question. For this reason we developed a new questionnaire. For the development of the questionnaire topics relevant to either the route via the nurse specialist or the usual care of the GP were identified in interviews with UI patients and experts in the field participating in the study. We developed a set of items to enable us to measure and test multi-items scales. The items are related to relevant themes with respect to care for UI patients according to standards on UI.

6. Evaluation of the availability and involvement of the nurse specialist by GPs will be measured by interviews and questionnaires after the study in a sample of the participating GPs. Data will be collected by a trained research assistant.

7. Evaluation by all participating nurse specialists on role participation will be measured by a semi-structured interview and questionnaires before, during and after the study.

Data collection of all outcome measures will be done at 3 and 12 months. For the cost-effectiveness study extra follow-up data collection of the primary outcome measure, costs and quality of life will take place at 6 and 9 months. In case of non-response, telephone reminders will take place two to three weeks after sending the questionnaires. Adverse events/effects will be monitored for the duration of the study.

### Sample size calculation and feasibility of recruitment

Based on a mean score on the ICIQ-UI SF [44] of 7.18 (sd 6.64), and an expected improvement of 2 on the outcome scale from 0 to 21 (which gives a delta value of 2/6.64 = 0.301), a power of 80% and a significance level of 0.05, and given the two-sided H1-hypothesis, that the new professional improves the effect, the needed number of patients per arm is 175 patients. We expect a drop-out rate during the trial of 20% and therefore we set our target at (rounded off) 2 times 210 = 420 patients. As shown in previous Dutch studies on UI, GP practices are able to include 5–10 patients per year. Therefore, we need 50 to 88 GPs to participate. Recruitment of the needed numbers of patients seems highly feasible since the power calculation was based on the regions of Maastricht and Nijmegen alone (total number of inhabitants: 500.000; total number of GP practices: approximately 240 GPs).

### Data-analysis and presentation/synthesis

Data collection, processing and analyses will be done with SPSS/PC, version 12 and 15. Variables will be analysed with the parametric Student-t test or the non-parametric U-test of Mann-Whitney (Wilcoxon ranksumtest) and the Wilcoxon-test for two unpaired samples. To compare the effects between different groups repeated measures AN(C)OVA and linear regression models will be used. Analyses will be done according to the intention-to-treat-principal. In case of missing values, non-compliance, loss-to-follow-up, drop-outs and protocol deviations also per-protocol analyses will be done for the economic evaluation only. To compare GPs using the nurse specialist and GPs who do not, per-protocol analyses will be performed.

### Economic evaluation

A cost-effectiveness analysis will be performed from the societal perspective. In this analysis the incremental health effects and the incremental costs of involving a nurse specialist in primary care for adult patients with UI, as compared to usual care, will be weighted. The major potential health effects of involving a nurse specialist are likely to result from an increase in the utilization of available therapies with proven effectiveness for UI, such as bladder training and/or pelvic floor muscle training. The EuroQol will be used to calculate quality adjusted life years (QALYs) following the algorithm developed by Dolan et al. [45]. Health care costs, productivity costs and patient and family costs will be included in the analysis. Health care costs (the use of diagnostics, treatment and incontinence pads) and productivity costs, time costs and travel costs will be collected using both registration systems and retrospective cost questionnaires. The cost questionnaire will be filled in by the patients at baseline, 3, 6, 9 and 12 months. Cost calculation will be based on real prices or on unit prices from the Dutch Guideline for Cost Calculation [46]. For the nurse specialist a cost price calculation will be performed. Costs associated with productivity loss will be calculated using the friction costs method [46]. In case of household, or other unpaid activities, shadow prices will be used. When clinical relevant effects of decreasing the impact of UI on daily life, and thus the use of incontinence pads, will be found during the one year of the study, a Markov type health state transition model will be used to calculate the long-term cost-effectiveness of the intervention [see Additional file 4].

## Discussion

In this paper we describe the study protocol of, to our knowledge, the first randomized controlled trial on care for UI patients in general practice that includes a full economic evaluation and cost-effectiveness modelling exercise from the societal perspective.

Next to this, despite the growing emphasis in the Netherlands on involving nurse specialists in general practice, specifically for UI this is still unfamiliar and the delegation of tasks to a nurse specialist can be considered as a new approach [17,47].

### (Potential) strengths of the study protocol

#### - The study design

The choice for a pragmatic design ensures that the intervention is as close as possible to treatment options in clinical practice (including 'cascades' of patient management choices). This makes implementation in the future easier. A care protocol with the preferred route via the nurse specialist is used. GPs will be well informed and stimulated to follow this care protocol. Despite this, there is a chance that GPs will not optimally use the nurse specialist because of perceptions of GPs or unfamiliarity with this new professional. For this reason perceptions such as willingness to use the nurse specialist, expectations, first experiences and reasons/promoting or hampering factors for GPs to use or not to use this route will be measured and actively monitored.

#### - Recruitment strategy

A particular strength is the recruitment strategy. Both new and already diagnosed UI patients are eligible. By recruiting already diagnosed UI patients we are likely to also include the majority of UI patients who mainly use pads and did not receive adequate conservative treatment before. In addition, this approach will be helpful to include the required number of patients in the available time period.

#### - Randomization approach

A third strength of our study protocol is the randomization approach, in which allocation is concealed and done by an external independent person. Randomization is done on patient level. An alternative would be that GPs would be randomized instead of patients. As GPs in the control group probably will have difficulties to include enough patients, this alternative seemed unfavourable.

#### - Competences of the nurse specialists

Another strength of our study protocol is that all nurses are trained and assessed in a uniform way to assure that the intervention will be carried out conform the intervention protocol in all four regions. To be able to get insight into their actual performance, all nurses will register their actions during all patient visits. This registration will be used to search for factors related to the intervention process that might influence the effectiveness of the intervention. It may also show an individual learning curve, as this intervention is new for the nurses.

#### - Outcome measurements

Diagnosing the type of UI is primarily done by the GP. Since we really want to mimic routine practice as close as possible we did not choose for objective measurements to confirm the diagnose of the type of UI by urodynamic testing. Neither did we choose for a pad test to measure the severity of UI. On the one hand because we felt that a pad test would be too much of a burden especially for the control group only receiving usual care. On the other hand because a pad test may act as a feedback which might influence the usual care too much. Furthermore, in the analyses by taking the baseline scores into account the information bias resulting from subjective self-reports will be reduced. Moreover, the self-reported data on healthcare use and costs will be validated by data from external sources like insurance companies (patients will give their informed consent).

### (Potential) limitations of the study protocol

#### - Participant selection

Selection of participants may limit generalization of the results of this study as selective non-response of patients as well as selective drop-out and selective refusal of GPs are possible.

Selective refusal of patients to participate might be assumed as some patients label UI as an accepted aspect of normal aging or do not experience adverse consequences. Possibly, such UI patients will refuse more often to participate than other UI patients. The same might be assumed about the more complex UI patients, who are severely burdened by multi-morbidity, and might be afraid to become even more burdened with participating in the study measurements. To limit such selective refusal patients living in care – and nursing homes are excluded from the study. To prevent selective drop out of patients in poor health states, the questionnaires are designed to minimize completion burden. Selective refusal of GPs to participate is anticipated from GPs already working with (in)continence nurses or specialised physical therapists.

Randomization on patient level could lead to contamination, and bias the results of this study. However, the influence of contamination is minimised, because patients in the usual care group have no access to the intervention of the nurse specialist. Nevertheless, it is possible that participating GPs are encouraged by the study to give more attention to the UI patients participating in the usual care group. As a result our findings may be conservative.

## Conclusion

The study will provide evidence whether the availability of a nurse specialist in a new role as substitute for the general practitioner leads to more effective and efficient care for adult urinary incontinence patients. Furthermore, the results will show whether this availability improves the quality of life of patients and the satisfaction of patients, general practitioners and other care providers. If this intervention proves to be effective, implementation of this intervention is considered and anticipated.

First results of this study will become available autumn 2008.

## Competing interests

The author(s) declare that they have no competing interests.

## Authors' contributions

All authors have read and approved the final manuscript. PAH is the researcher and first author of this manuscript; is involved in the design of the study, data collection, statistical analysis, the development of the experimental intervention; training and assessment of the competences of the nurse specialists. BB is together with RW principal investigator, wrote the study protocol, is involved in the training of the nurse specialists, has made substantial contributions to the development of the experimental intervention, supervises the planning and project and is involved in revising the article for important intellectual content. MJ was helpful in writing the study protocol, supervises the planning and project, wrote the economic evaluation part of the study protocol and supervises the economic evaluation and is involved in revising the article for important intellectual content. TLJ was helpful in writing the study protocol, supervises the planning and project; is involved in the training and assessment of the competences of the nurse specialists, has made substantial contributions to the development of the experimental intervention and is involved in revising the article for important intellectual content. JS gave advice regarding the study protocol, supervises the economic evaluation, the planning and project and is involved in revising the article for important intellectual content. FN was helpful in writing the study protocol, is responsible for methodology and the statistical analysis; performed the power calculation and is involved in revising the article for important intellectual content. RW is together with BB principal investigator, wrote the study protocol and supervises the planning and project; is involved in the assessment of the competences of the nurse specialists and is involved in revising the article for important intellectual content.

## Pre-publication history

The pre-publication history for this paper can be accessed here:



## Supplementary Material

Additional file 1 Training and competences of the nurse specialist. This document explains the training and competences of the nurses prior to and during the study. Click here for file

Additional file 2 Intervention nurse specialist: diagnostic process, evaluation and therapeutic process. This document gives a detailed description of process of the intervention given by the nurse specialists. Click here for file

Additional file 3 Description intervention nurse specialist. This document gives a detailed explanation of the information and advices on urinary incontinence given by the nurse specialists. Click here for file

Additional file 4 Modelling approach. This document explains how the long-term cost-effectiveness of the intervention will be calculated when clinical relevant effects of decreasing the impact of UI on daily life, and thus the use of incontinence pads, will be found during the one year of the study. Click here for file
