# Supplementary material for: The effects of involving a nurse practitioner in primary care for adult patients with urinary incontinence: The PromoCon study (Promoting Continence)
Source: BMC Health Serv Res. 2008 Apr 15;8:84. doi: 10.1186/1472-6963-8-84 (PMC2386786; doi:10.1186/1472-6963-8-84)
Supplement: Additional file 1 — Training and competences of the nurse specialist. This document explains the training and competences of the nurses prior to and during the study. [file 1472-6963-8-84-S1.doc]

**Additional file 1 Training and competences of the nurse specialist**

The intervention means incorporating a nurse specialist to support the GP, by taking care of the diagnostic and therapeutic management (based on guidelines) of patients with the most prevalent types of UI (stress UI, urgency UI and mixed UI).

During two days of 7 hour sessions with at least one week interval, each participating nurse will be trained by specialists in the field of UI (a general practitioner, a pelvic floor therapist and a continence nurse), how to take over tasks related to diagnostics, intervention and monitoring of incontinence from the GP based on guidelines and protocols. As mentioned earlier, UI is especially a problem for elderly women. In this population group the problem is often more complex and concurrent co-morbidities related to UI result in discomfort and handicap [1]. So, almost automatically, the higher prevalence, nature and severity of the health problem will affect the work of the nurse specialist. During the training of the nurse specialist, special attention will be given to the special needs of this population group. The same applies for the potential impact of cultural background.

In between and after the training days, the nurses have to practice their newly learned skills on pilot patients, will visit an experienced continence nurse, a specialized pelvic floor therapist and a urology department to get insight in demarcation of the different specialties dealing with UI patients. Next to this they will have to spend time to prepare and organize the necessary materials and accommodation for the intervention. Finally, in a two and half hour session each individual nurse must prove her competence both in an individual written assessment and an assessment with a simulated patient. Because this is a new intervention, before and during the time of the study all participating nurse specialists will meet on a regular base for intervision both with each other and specialists in this field of the research team. These specialists can be contacted to discuss cases at any time. Since this nurse specialist is a new profession a learning curve is to be expected.

1. Lagro-Janssen ALM, Teunissen D, Breedveldt-Boer HP, Dongen van JJAMv, Lemain TJJ, Steeneken F, Dijkstra RH, Wiersma TJ**: NHG-Standaard Incontinentie voor Urine M46 Eerste herziening (Dutch College Guideline on Urinary Incontinence. First revisio**n)*. Nederlands Tijdschrift voor Urologie 3, 65-7*2 2006**,** 49(2):501-510.
