# Supplementary material for: The effects of involving a nurse practitioner in primary care for adult patients with urinary incontinence: The PromoCon study (Promoting Continence)
Source: BMC Health Serv Res. 2008 Apr 15;8:84. doi: 10.1186/1472-6963-8-84 (PMC2386786; doi:10.1186/1472-6963-8-84)
Supplement: Additional file 2 — Intervention nurse specialist: diagnostic process, evaluation and therapeutic process. This document gives a detailed description of process of the intervention given by the nurse specialists. [file 1472-6963-8-84-S2.doc]

**Additional file 2 Intervention nurse specialist: diagnostic process, evaluation and therapeutic process**

First visit:

##### Diagnostic process

Input: presumed diagnosis GP with relevant information; copy of baseline data and measurements.

Standardised history taking: registration of problems in pelvic floor/bladder function (impairment), activities (disability) and participation problems and the influence of personal and external factors. (International Classification of Functioning, Disability and Health-ICF). Check bladder diary.

##### Evaluation

Classifying type of incontinence: stress-, urgency- or mixed urinary incontinence.

Treatment plan: the nurse specialist discusses and provides relevant information to the patient about the treatment plan, with respect to the diagnosed problem and the cognitive and physical possibilities of the patient. She points out her task and what is expected from the patient. The nurse specialist can use bladder diaries and 24 hours home pad tests during daily activity as evaluation and feedback instruments.

##### Therapeutic process

The nurse specialist follows the next more or less standardised treatment plan based on best available evidence.

1. Information, education and advice on anatomy and function of bladder and pelvic floor muscles, good toilet behaviour, influence lifestyle and, when appropriate, mobility, co-morbidity and cognition on urinary incontinence.

2. When bladder diary abnormal: continue first with advice on bladder training; repeat diary. When bladder diary not improved after 3 weeks: consult GP. When bladder diary normal: continue with advice on pelvic floor muscle (PFM) function.

3. Assess the functional possibilities of the PFM of the patient: the patient checks at home awareness of PFM function: a) contraction: ability to postpone micturition for maximally 10 seconds; b) relaxation: ability to initiate and continue micturition with a good flow without hesitation and/or interruption.

Follow-up visit(s):

4. Patient is aware and in control over this function of the PFM: continues advice on adequate use of PFM function and training. Patient is not aware or in control of the PFM: the nurse specialist consults the GP to check PFM function by digital vaginal and/or rectal palpation.

Awareness and control improved: the nurse specialist continues PFM training (PFMT).

Still no or insufficient awareness and/or control after check GP or after 6 weeks: the nurse specialist consults the GP.

5. Advice on incontinence pads or other non-curative materials adjusted to individual needs and wishes of the patient and to prevent problems as a result of the used materials. The main goal of the nurse specialist is to check whether the use of the means and pads is needed or can be reduced by information, advice and bladder and/or PFMT.

6. Effect on urinary incontinence after 3 months: insufficient improvement or failure (no cure): consult GP. Sufficient improvement or cure: follow-up patients to stimulate compliance and prevent fall back and thereby improving the long term effect of the intervention during the first year after start of the intervention. Patients can either contact the nurse specialist when they experience fall back or are actively contacted by the nurse specialist at 6 and 12 months after start of the intervention. In case of fall back he or she considers together with the patient a consult with the GP for a refresher intervention.
