# Supplementary material for: The effects of involving a nurse practitioner in primary care for adult patients with urinary incontinence: The PromoCon study (Promoting Continence)
Source: BMC Health Serv Res. 2008 Apr 15;8:84. doi: 10.1186/1472-6963-8-84 (PMC2386786; doi:10.1186/1472-6963-8-84)
Supplement: Additional file 3 — Description intervention nurse specialist. This document gives a detailed explanation of the information and advices on urinary incontinence given by the nurse specialists. [file 1472-6963-8-84-S3.doc]

**Additional file 3 Description intervention nurse specialist**

**Information, education and advice** about

- anatomy and function of the pelvic floor and pelvic organs in general and specific with activities of daily life (weight bearing possibilities of the pelvic floor)

- - healthy toilet behaviour (relate to findings bladder diary)
  - lifestyle influence on incontinence: weight control, smoking, dietary factors (fluid intake: relate to bladder diary), constipation, postural changes, hygiene, etc. (the load)
  - the role of co-morbidity, dexterity and mobility, cognition and aging, hormonal status

**Check bladderdiary** on normal toiletbehaviour: micturition daytime 5-7 times, night time 0-2 times with normal fluid intake 1½ -2 litres daily. Normal maximal voided volume for adults varies from 300-700 cc, control mean voided volumes. Relaxed micturition without straining.

**Bladdertraining** mainly based on the signs and symptoms and the diagnostic procedures such as the bladder diary. Goal of bladder training is normalising frequency of micturition. When patients go infrequent or irregular stimulate to go more frequent with regular 2-3 hour voiding intervals. When frequency is over 7 times daily then, on the basis of extrapolation from the bladder retraining literature, the outpatient retraining protocol includes an initial voiding interval typically beginning at 1 hour during waking hours, which is increased by 15-30 minutes per week depending on tolerance of the schedule (i.e., fewer incontinent episodes than the previous week, minimal interruptions to the schedule, and the patients feeling of control over urgency), until a 2-3 hour voiding interval is achieved. A shorter initial voiding interval, i.e., 30 minutes or less, may be necessary for patients whose baseline urinary diaries reveal an average voiding interval of less than 1 hour. Education will be provided about normal bladder control and methods to control urgency such as distraction and relaxation techniques and pelvic floor muscle contraction. Self-monitoring of voiding behaviour using bladder diary and treatment log will be included in order to determine adherence to the schedule, evaluate progress, and determine whether the voiding interval should be changed. The nurse specialist monitors progress, determines adjustments to the voiding interval, and provides positive reinforcement to patients undergoing bladder retraining weekly (minimum 3 weeks 20 minutes visits) during the training period (follow-up 15 minute visits at 6 weeks, 3, 6 and 12 months).

**Control and advice on PFM function and PFMT:** since legal restraints do not allow the nurse specialist to examine the actual PFM she/he has to give an exercise advice based on the patients observations of contraction and relaxation as formulated in the first visit. If the patient is able to do so explanation about the functional use and training of the PFM as follows:

Stress urinary incontinence: PFM must be contracted to improve closure of the urethra whenever the pelvic floor is loaded either by coughing, lifting, jumping and with activities where the patient experiences urinary incontinence. Contractions may be quick and short or prolonged with power depending on the activity.

Urgency urinary incontinence: the PFM is used to inhibit detrusor overactivity, this can be short and quick or prolonged.

In case of mixed incontinence PFMT is used for both goals with inhibition of detrusor overactivity as the first goal. The nurse specialist monitors progress, determines adjustments to the PFMT, and provides positive reinforcement to patients undergoing PFMT weekly (minimum 3 weeks 20 minutes visits) during the training period (follow-up 15 minute visits at 6 weeks, 3, 6 and 12 months).

**Overview treatmentplan**

| Stress urinary incontinence   - Toiletbehaviour - PFMT: improve closure mechanism - If necessary: advice means / materials | Urgency urinary incontinence   - Bladdertraining - PFMT: inhibition bladderactivity - If necessary: advice means / materials | Mixed urinary incontinence   - Bladdertraining - PFMT: inhibition bladderactivity - PFMT: improve closure mechanism   If necessary: advice means / materials |
| --- | --- | --- |
