# Supplementary material for: The effects of involving a nurse practitioner in primary care for adult patients with urinary incontinence: The PromoCon study (Promoting Continence)
Source: BMC Health Serv Res. 2008 Apr 15;8:84. doi: 10.1186/1472-6963-8-84 (PMC2386786; doi:10.1186/1472-6963-8-84)
Supplement: Additional file 4 — Modelling approach. This document explains how the long-term cost-effectiveness of the intervention will be calculated when clinical relevant effects of decreasing the impact of UI on daily life, and thus the use of incontinence pads, will be found during the one year of the study. [file 1472-6963-8-84-S4.doc]

# Additional file 4 Modelling approach

When clinical relevant effects of decreasing the impact of UI on daily life, and thus the use of incontinence pads, will be found during the one year of the study, a Markov type health state transition model will be used to calculate the long-term cost-effectiveness of the intervention.

The population in the model consists of patients who consult(ed) their GP with complaints of UI. Relevant health states for the model will be defined, and patient time spent in these different health states will be calculated. Time spent in the health states will be weighted against both generic quality of life (EuroQol) and experiencing incontinence specific problems (ICIQ). Cost-effectiveness acceptability curves will be determined to indicate the probability of cost-effectiveness given varying levels of the society’s willingness to pay for a QALY. All model parameters will be based on outcomes observed in the underlying study or an extensive literature review. When no data is available, or when the data is insufficiently reliable, parameter estimates will be derived from panel sessions with leading experts in the field. Cycle length will be one month. One way sensitivity analysis will be performed to establish the separate effect of model parameters on the results of the analysis. The model parameters will be varied across a plausible range. Parameter uncertainty will be further tested using probabilistic sensitivity analysis. Probabilistic sensitivity analysis takes into account the fact that some combinations of factors are more likely to take place than others. The population in the model will be followed until the last patient has deceased. Excel spreadsheet modelling will be used to perform the calculations. Future costs and effects will be discounted according to Dutch guidelines [1].

1. Oostenbrink JB, Bouwmans CM, Koopmanschap MA, Rutten FFH**: Manual for Cost Analys**is. Amstelveen: College voor Zorgverzekeringen. (In Dutch); 2004.
